# A root cause analysis of sub-optimal uptake and compliance to iron and folic acid supplementation in pregnancy in 7 districts of Zambia

**DOI:** 10.1186/s12884-019-2700-1

**Published:** 2020-01-06

**Authors:** Moses C. Simuyemba, Phoebe A. Bwembya, Mumbi Chola, Charles Michelo

**Affiliations:** 1 Department of Community and Family Medicine, University of Zambia School of Public Health, Lusaka, Zambia; 2 Department of health Policy and Management, University of Zambia School of Public Health, Lusaka, Zambia; 3 University of Zambia School of Public Health, Lusaka, Zambia

**Keywords:** Iron supplementation, Folic acid supplementation, Zambia, Root cause analysis, Anaemia and pregnancy, Antenatal

## Abstract

**Background:**

Iron and Folic Acid are two of the micronutrients recommended for pregnant women to support optimal maternal outcomes with regard to preventing anaemia and foetal birth defects. It is difficult to establish if women provided with iron and folic acid supplementation in Zambia benefit from it and how well it is implemented. The overall objective of this study was to determine the levels of uptake and compliance to iron and folic acid in pregnancy among women of child-bearing age in Zambia, with a focus on both supply and demand factors.

**Methods:**

A cross sectional, mixed method study was done. Data was collected in August and September 2015 from six of the 14 districts in which Scaling Up Nutrition interventions were being undertaken as well as Lusaka district. A household survey covering 402 males and females of child-bearing age, 27 key informant interviews amongst key stakeholders and 12 focus group discussions at community level were conducted.

**Results:**

Antenatal clinic attendance was almost universal (98.7%); the majority of both men (92.1%) and women (97.4%) had heard messages about iron and folic acid supplementation; the majority (96.5%) of women reported having taken iron and folic acid tablets during their last pregnancy, with 61.3% starting in the second trimester, 27.2% during the first trimester, and 7.7% in their third trimester. Eighty-five per cent (80.5%) of the women reported that they had taken all the tablets they were given with about 13.4% not taking all the tablets received.

**Conclusions:**

Root cause analysis, using both qualitative and quantitative findings, showed that the main challenges faced were long distances to health facilities and high transport costs; some women not being reached with supplementation messaging; lack of formalised and uniform training around delivery of antenatal messages across health care workers; women not attending antenatal monthly to replenish supplements; and forgetfulness to take the drugs daily. While male involvement may be a supportive factor, it sometimes hinders women from accessing antenatal services. Results showed that both uptake and compliance to iron and folic acid supplementation in pregnancy in Zambia were sub-optimal.

## Background

In Zambia, anaemia is one of the major health problems faced by women during pregnancy. It may arise due to insufficient iron intake and conditions such as malaria or worm infestation. In women of reproductive age, haemoglobin levels of less than 120 g/L are an indication of anaemia [1]. According to National Food and Nutrition Commission (NFNC), haemoglobin concentrations of < 120 g/L were found in 29.1% (95% CI: 24.7,33.4) of women of reproductive age, indicating moderate anaemia [2]. Lubeya and Vwalika [3] found that 36.2% of women in their study in Lusaka, Zambia, had anaemia during pregnancy. In a systematic review of 29 cohort studies in low- and middle-income countries, Rahman et al. [4] found that 42.7% of women experienced anaemia during pregnancy. The World Bank [5] puts the prevalence of anaemia in pregnancy worldwide at 40.1% and at 39.1% in Zambia. From these findings, it can be said that Zambia has a high prevalence of anaemia in pregnancy.

For women, iron deficiency contributes to decreased productivity [2] increased occurrence of intrauterine growth retardation, low birth weight and perinatal mortality [6]. Daru et al. [7] found that maternal death was up to 2.36 (95% CI 1·60–3·48) times higher in women with severe anaemia compared with those without severe anaemia.

Iron and Folic Acid are two of the micronutrients recommended for women planning to become pregnant to support optimal maternal outcomes [8]. In Zambia, the ministry of health (MOH) advises pregnant women to take an iron tablet daily throughout their pregnancy and lactating period, starting from the second trimester and continuing to 45 days after childbirth [9]. Current recommendations support supplemental intake of iron (60 mg/day) and folic acid (0.4 mg/day) throughout the gestational period for optimal maternal outcomes [10]. However, supplementation with folic acid is not beneficial in preventing birth defects if commenced after 3 months of pregnancy.

Even though the Zambian policy focuses on promoting iron and folic acid during pregnancy, in India, positive birth outcomes regarding improvements in weight, length, head circumference and chest circumference were observed among children from mothers who were supplemented with vitamin B12, in addition to folic acid [11]. Current literature also suggests that in addition to enhancing the absorption of nonheme, vitamin C is important in the regulation of cellular iron uptake and metabolism [12]. Vitamin C has also been found to be critical in the absorption of iron from supplements, particularly in situations where low serum levels are common [13]. Based on emerging discourse, consideration beyond the provision of iron and folic acid should be considered in the prevention of anaemia.

In acknowledging the high undernutrition rates, Zambia has endorsed the campaign for Scaling Up Nutrition (SUN) interventions. Policy initiatives are stated in the Food and Nutrition Policy, defining areas of attention for nutrition programs [14]. The global campaign on Scaling Up Nutrition (SUN) has identified anaemia as one of the entry points for promoting maternal health and iron and folic acid have been proposed as two of the important micronutrients to include in the interventions for the SUN, hence the focus of this study. The Department of Public Health at the University of Zambia, with support from the Scaling Up Nutrition (SUN) fund managed by Care international, undertook a research to provide insights into how compliance and early uptake of iron and folic acid could be improved. This was important to inform policy on adopting supplementation strategies that support women for optimal maternal outcomes and improved child nutrition.

### Objectives

The overall objective was to determine the uptake and compliance to iron and folic acid in pregnancy among women of child-bearing age in Zambia.

Specific objectives of the study were:

1. To establish the current levels of compliance and uptake of iron and folic acid supplementation and establish associated demographic and socio-economic factors.
2. To determine the supply-side constraints associated with early uptake and compliance to folic acid and iron in pregnancy.
3. To assess available capacity among health care staff for supporting folic acid and iron supplementation in pregnancy.
4. To assess messages disseminated on folic acid and iron for pregnancy, their formats, and appropriateness.

## Methods

A cross sectional, mixed method study was conducted in August and September 2015. Data was collected from six of the fourteen districts in which SUN interventions were being undertaken, namely Kasama and Kaputa (Northen Province), Chipata and Lundazi (Eastern Province) and Mongu and Shangombo (Western Province). Lusaka, being the location of head offices for all government ministries, agencies and many civil society organisations, was included to make a total of seven districts for the study. Districts were purposively selected to capture both rural and urban communities.

To collect knowledge, attitude and practice data, a household questionnaire was administered among men and women in selected communities from the six districts. All women and men aged 15–49 who were permanent residents of the households at the time of the study were eligible to be interviewed. However, the final random sampling only included participants 16 years and older. The survey employed multi-stage cluster sampling. It adopted sampling methods like those used in the Zambia demographic and health surveys. A stratified sample was selected in two stages. In the first stage, the standard enumeration areas (SEAs) from the 2010 Census of Population and Housing were selected with probability proportional to the standard enumeration area size. The household listing operation was conducted in all selected SEAs, with the resulting lists of households serving as the sampling frame for the selection of households in the second stage. Selected SEAs with more than 300 households were segmented, with only one segment selected for the survey with probability proportional to the segment size. Household listing was conducted only in the selected segment. Therefore, a cluster was either an SEA or a segment of the SEA. In the second- stage selection, an average number of 25 households was selected in every cluster, by equal probability systematic sampling. A complete listing of households was used to select the households. All private households were listed. The listing excluded people living in institutional households (army barracks, hospitals, police camps, boarding schools, etc.) as the context and dynamics tend to be different than in the general population.

To explain factors associated with anaemia among pregnant women, qualitative data was collected using key informant interviews (KIIs), focus group discussions and observations. Twenty-seven (27) KIIs with the various stakeholders were carried out, including Ministry of Health, Ministry of Community Development Mother and Child Health, Ministry of Local Government and Housing, National Food and Nutrition Commission, Medical Stores Limited, district health management teams and the health facilities. A total of 12 focus group discussions with community members were conducted with six in rural and six in urban areas for a total of 2 FGDs in each district. In areas where prominent individuals (headmen or chiefs) involved in the programme were present, in-depth interviews were conducted with them in order to fill any information gaps that were identified. Although not designed with them in mind, the study conformed to the “Standards for reporting qualitative research: a synthesis of recommendations” [15].

Quantitative data was analysed using STATA at 95% confidence interval and 5% margin of error, and thematic analysis was applied to qualitative data. Root cause analysis (RCA) was utilised to relate the findings from the qualitative and quantitative data. RCA is a process designed for use in investigating and categorizing the root causes of events and helps in identifying what, how and why something happened [16]. The process involves data collection, causal factor charting, root cause identification and recommendation generation, with implementation where appropriate [16]. Although implementation was beyond the scope of this work, the root cause analysis went further to explore and classify contextual factors, challenges, responses and consequences, as outlined in the modified methodology utilised by the Gavi Full Country Evaluation, FCE [17].

## Results

### Demographic and socio-economic factors

A total of 402 respondents (77.9% females and 22.1% males) participated in the household survey from a calculated sample size of 397. Results in Table 1 show the distribution of population characteristics.

**Table 1:** Distribution of Participants’ characteristics

Variable	Freq.	Percent
Age		
15–19	22	5.5
20–24	88	21.9
25–59	84	20.9
30–34	87	21.6
35–39	55	13.7
40–44	33	8.2
45–49	16	4.0
Don’t Know	17	4.2
	Mean (SD)	Min, Max
Age	29.8 (7.6)	16, 49
Sex	Freq.	Percent
Male	89	22.1
Female	313	77.9
Marital Status	Freq.	Percent
Married	319	79.4
Living Together	2	0.5
Divorced	22	5.5
Separated	6	1.5
Widowed	3	0.8
Never Married	50	12.4
Education Level	Freq.	Percent
Primary	163	40.6
Secondary	188	46.8
Tertiary	8	2.0
None	41	10.2
No Response	2	0.5
Province	Freq.	Percent
Eastern	196	48.6
Western	97	24.1
Northern	110	27.3
District	Freq.	Percent
Chipata	117	29.0
Lundazi	79	19.6
Kaputa	22	5.5
Nsama	11	2.7
Kasama	64	15.9
Mongu	22	5.5
Sioma	51	12.7
Shamg’ombo	23	5.7
Limulunga	14	3.5
Ever Pregnant	Freq.	Percent
Yes	312	99.7
No	1	0.3
Parity	Freq.	Percent
0	8	2.6
1	65	20.8
2	67	21.4
3	59	18.9
4	39	12.5
5	32	10.2
6	23	7.4
7	8	2.6
8	5	1.6
9	3	1.0
11	1	0.3
No Response	3	1.0
ANC Attendance	Freq.	Percent
Yes	309	98.7
No	2	0.6
No Response	2	0.6
Partner ANC Attendance	Freq.	Percent
Sometimes	186	59.4
All The Time	29	9.3
Never	93	29.7
No Response	5	1.6
Last Pregnancy	Freq.	Percent
Currently Pregnant	44	14.1
1 Year Ago	112	35.8
2 Years Ago	49	15.7
3 Years Ago	38	12.1
More Than 3 Years Ago	69	22.0
No Response	1	0.3
ANC Attendance Trimester	Freq.	Percent
First Trimester	87	27.8
Second Trimester	199	63.6
Third Trimester	21	6.7
Don’t Know	3	1.0
No Response	3	1.0
ANC Attendance Frequency	Freq.	Percent
1	15	4.79
2	20	6.4
3	93	29.7
4	105	33.6
More than 5	71	22.7
No Response	9	2.9

In the survey, age ranged between 16 and 49 years with the oldest respondent was 49 years old while the youngest was 16 years old. The mean age was 29.8 years, with a standard deviation of 7.6. Majority, 79.4%, of the respondents reported being married, while 12.4% had never been married. Approximately 6% reported that they were divorced. With regard to education level, 40.6% reported having primary education while 46.8% had secondary education. Only 2% had tertiary education whereas 10.2% reported having had no education.

The distribution of respondents across the three provinces in the study was as follows: Eastern Province 48.6%, Western Province 24.1% and Northern Province 27.3%. Distribution by district was such that in Eastern province, Chipata and Lundazi accounted for 29.0 and 19.6% of the entire sample respectively. From the Western Province, Mongu accounted for 5.5%, Sioma 2.7%, Shang’ombo 15.9% and Limulunga 5.5%. In the Northern Province, Kaputa accounted for 6.3%, Nsama 3.1% and Kasama 15.5%.

Almost all female respondents (99.7%) reported ever being pregnant with less than 1% reporting never having been pregnant. Of those who assented to “ever being pregnant”, 20.8% had one child, 21.4% had two children, 18.9% had three children, 12.5% had four children while 10.2% had five. The rest had more than 5 children.

Antenatal Clinic (ANC) attendance was almost universal (98.7%) with 59.4% reporting that they “sometimes” attended with their male partners. The majority (63.6%) of women attended ANC in the second trimester and only 27.8% attended in the first trimester. Regarding frequency of attendance, slightly over one third (33.6%) attended ANC 4 times while 29.7% had attended ANC 3 times. About 23% attended ANC more than 4 times.

There was no significant difference in ANC attendance among the different age groups or by district. However, there was a significant association (*p* = 0.001) between ANC attendance among women and parity using Fishers exact test. This suggests that women who have had children before were more likely to attend ANC. Significant observations were also noted between male partner attendance and initiation of ANC in the first trimester (*p* = 0.033) as well as frequency of ANC attendance (*p* = 0.001). Thus, women who attended with partners were more likely to attend ANC in the first trimester; and were more likely to have more antenatal visits.

### Uptake and compliance to folic acid and Iron supplementation

Regarding uptake and compliance to iron and folic acid supplementation, the results are presented in the Table 2 below.

**Table 2:** Iron and Folic Acid uptake and Compliance (*n* = 313)

Variable	Freq.	Percent
Took Iron and Folic Acid		
Yes	302	96.5
No	8	2.6
No Response	3	1.0
First Took Iron and Folic Acid		
First Trimester	85	27.2
Second Trimester	192	61.3
Third Trimester	24	7.7
No Response	12	3.8
Iron Tablets Source		
Health Facility	299	95.5
Community Health Worker	1	0.3
Bought from a Pharmacy	0	0.0
Relative	0	0.0
Friend	1	0.3
Other	1	0.3
No Response	11	3.5
Type of Iron Tablets		
Coated	277	89
Non-coated	17	5
No Response	19	6
Took All Iron Tablets Given		
Yes	252	80.51
No	42	13.42
No Response	19	6.07
Took All Folic Acid Tablets Given		
Yes	243	77.6
No	38	12.1
No Response	32	10.2

The majority (96.5%) of women reported that they had taken iron and folic acid tablets during their last pregnancy, with 61.3% of them taking the iron for the first time in their second trimester. Almost all (95.5%) of the women obtained their iron tablets from the health facility and 88.5% of the women reported receiving coated, non-slow-release, tablets. About 80 % of the women reported that they had taken all the iron tablets they were given with 77.6% of women taking all the folic acid tablets received. There were no significant associations noted between taking of the supplements and age, marital status, education or parity.

### Messages on Iron and folic acid

The majority of both men (92.1%) and women (97.4%) interviewed had heard messages about iron and folic acid supplementation. Most of the participants (90%) received the messages through the health facility. Nearly all women (97%) and 72% of males indicated that they heard messages on iron and folic acid supplementation from health facility staff. The most common message received by both men and women during ANC was on anaemia in pregnancy and about 92% of females and 85% of males had heard messages on anaemia in pregnancy. Messages on malaria in pregnancy and family planning were among the messages least heard. Nonetheless, the majority (87% women and 75% men) reported that enough time was taken to explain the messages they received. The majority of both men (62.8%) and women (70.2%) had reported understanding the messages very well.

## Discussion

Almost all women in the household survey (97%) reported that they had taken iron and folic acid tablets during their last pregnancy and most (61%) commenced in the second trimester (with 27% in first trimester and 8% in third trimester). It thus seems that reported uptake is high, though commencement is late, a situation which may lead to profound negative maternal outcomes. However, considering the qualitative findings which follow together with these quantitative findings, the study found that there was suboptimal uptake as well as compliance to iron and folic acid supplementation amongst pregnant women in the seven districts.

**Fig. 1:**
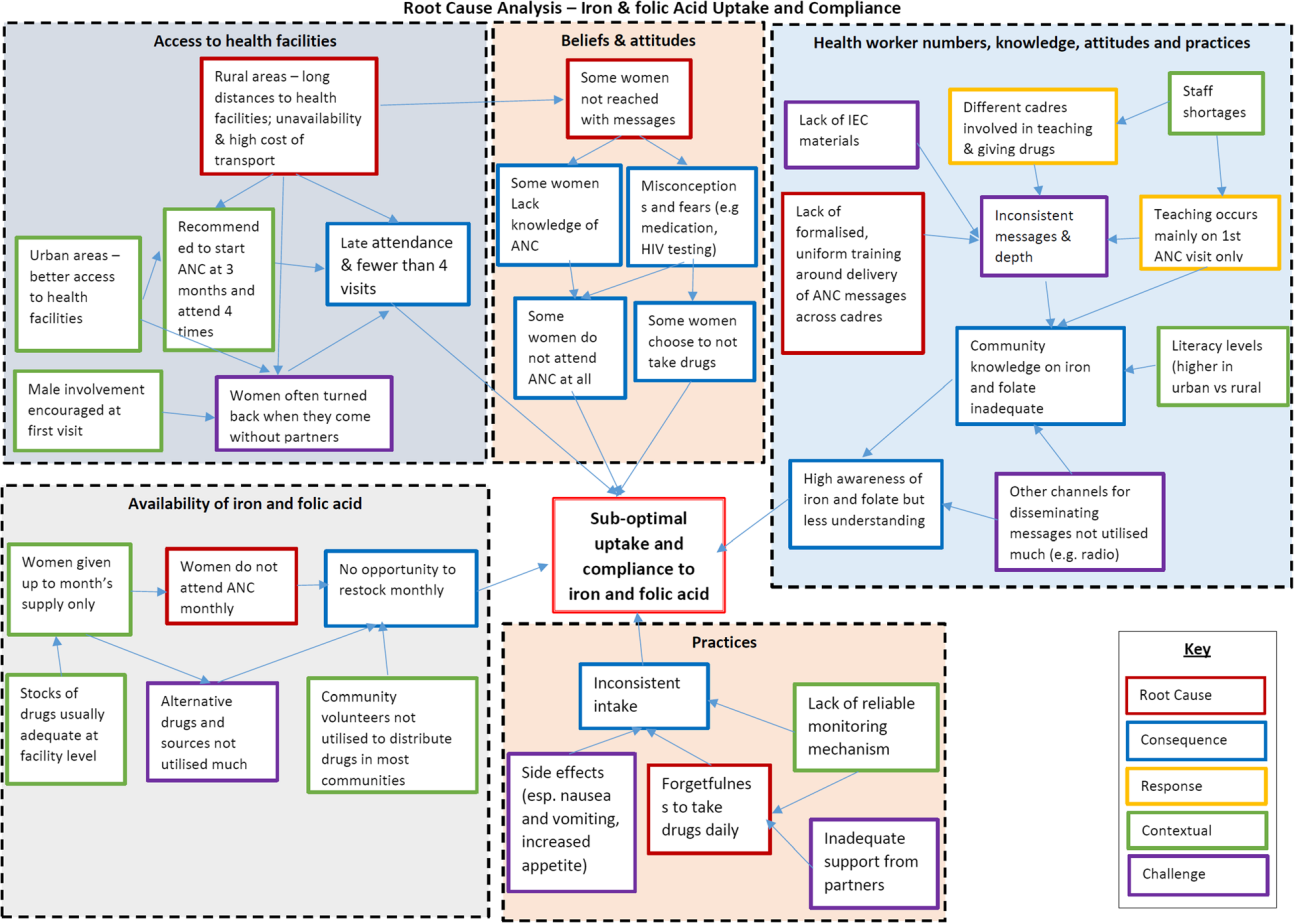
Overall root cause analysis

In discussing factors associated with this observation, a root-cause analysis (RCA) diagram in Fig. 1, adapted from Rooney and Heuvel [16] was applied. The overall root cause analysis diagram shows that access to iron and folate was hampered by numerous factors including beliefs and attitudes; health worker numbers, knowledge, attitudes and practices; availability of iron and folic acid; and certain practices. The root-cause analysis shows that the root causes of this underutilisation of iron and folic acid were long distances to health facilities coupled with unavailability and high cost of transport; some women not being reached with messages on ANC and iron and folate supplementation; lack of formalised, uniform training around delivery of ANC messages across health cadres and volunteers; women not attending ANC monthly whereas supplements were given as a monthly supply at a time; and not taking the supplements daily. These factors are discussed in more detail, using the RCA format that follows.

### Access to health facilities and ANC

#### Distance to health facilities and transport challenges

Distance to health facilities was a major barrier to accessing antenatal services and therefore iron and folic acid supplementation. This was worse for rural women who had to travel long distances to access health services. Urban areas fared better as more health facilities were available, usually within walking distances or reached by bus. Many women in rural areas had to walk for hours to reach health facilities or they had to hire transport, usually motorbikes, at a huge cost. Considering their economic situation, such challenges impeded receiving timely ANC services.

Other studies have also shown that rural women attend ANC less than urban women and also initiate visits later due to such barriers [18]. A study by Escamilla et al. [19] in rural Zambia found that for every kilometre increase in distance from the household to the health facility, the association with PMTCT regimen uptake and combination antiretroviral regimen uptake decreased. In other studies in Zambia, distance to a health facility was a key factor in determining whether a woman delivered at home or at a health facility [9, 20]. Impediments related to distance were linked to other costs that might be incurred, such as transport, lodging and food [9, 20].

In this study, women were also anxious about additional costs associated with long distances to health facilities such as food and lodging. These discouraged them from going for ANC, especially when it was suspected that they would be requested to remain at the health facility until they gave birth.

*“…coming for antenatal, I really want to attend but because of not having money I am unable to attend antenatal.”* Key informant.

In supporting women to attend antenatal clinics, Escamilla et al. [19] recommended program models that decentralize care into remote communities as one way of dealing with this challenge.

#### Initiation of antenatal care visits

The recommendation in the places visited was for women to start their ANC visits at 3 months of pregnancy rather than as soon as they knew they were pregnant. At the time of the study, officially in Zambia, antenatal visits were recommended by the end of 16 weeks of pregnancy and at 24, 32 and 36 weeks, except for women with complications [21].

*“Ideally, they are supposed to come as early as 12 weeks but we are having a challenge. We have involved some SMAGS, safe motherhood action groups, to be helping us sensitize the women but all the same women who come at 12 weeks the number is still very low. Most of them come in the second trimester.” Nurse KI*

Nonetheless, focus group discussions reflected that most women interpreted the recommendation to mean that they should report to ANC at or after 3 months. Miscommunication about when to report for antenatal might be a factor contributing to pregnant women not receiving comprehensive antenatal care.

Three months’ gestation is late to get the full benefits of folic acid supplementation with regard to prevention of neural tube defects. These occur in the first few weeks of pregnancy whilst the baby’s organs are being formed [6, 22]. Current literature recommends that folic acid be given pre-conception for maximum benefit. Some countries have adopted fortification of food such as flour with folic acid in order to increase pre-pregnancy levels of folic acid in women of child-bearing age [10]. Although the benefits of increasing haemoglobin levels might have been realized, women in this study were most likely not getting the full benefit of folate supplementation for optimal foetal outcomes.

#### Male involvement in antenatal care

This study demonstrated that male involvement encourages both early attendance and frequency of attending ANC. The merits of male involvement in ANC have been investigated in other studies in Zambia [23, 24] found that women who were accompanied by their male partners for ANC delivered at health facility more than those who did not. Additionally, a greater proportion of such women returned for postnatal care. Male involvement was thus observed to have been essential in contributing to improved ANC and PNC attendance [23].

National policy on male involvement encourages women to bring their partners along for ANC, especially for the first antenatal visit. However, in a lot of cases, male involvement was misinterpreted by health facility staff to mean that women who did not come with partners should not be attended to. Thus, many women were turned away at some health facilities as the nursing staff insisted on them coming to ANC with partners. As a result, those who were unable to come with their partners did not come at all or they delayed coming for their first visit until their partners were available. This situation adversely affected women who were pregnant outside wedlock.

In some cases, women were asked to come with letters from traditional leaders to explain why they could not come with their partners and in other cases they were forced to just bring any man with them so that they were attended to. Sometimes, pregnant women had to pay for such letters from traditional leaders to exempt them from bringing their male partners. The male partners were not available in some cases due to work or they were afraid to be tested for HIV during the first antenatal visit. Coupled with long distances to health facilities in rural areas the faulty application of the male involvement policy has become a major barrier to accessing antenatal services and iron and folic acid supplements.

*“If you have not come with your husband, they chase you telling you to come with your husband”. FGD participant.*

*“When your husband is not around and they send you back, we get any man to accompany us.” FGD participant*

This placed an extra burden on women attending ANC and made male involvement become an unintended barrier despite its good intentions. The fear among men of routine investigations conducted at ANC, including for sexually transmitted infections (STIs) and Human Immunodeficiency virus (HIV), was also documented in a similar study in Uganda [18] and may point to a greater need to educate men about ANC and health care services in general.

*“Maybe he has HIV and the wife does not have so he becomes afraid.” Nurse KI*

Sometimes, HIV testing was a discouragement for attending ANC even amongst some women.

**Fig. 2:**
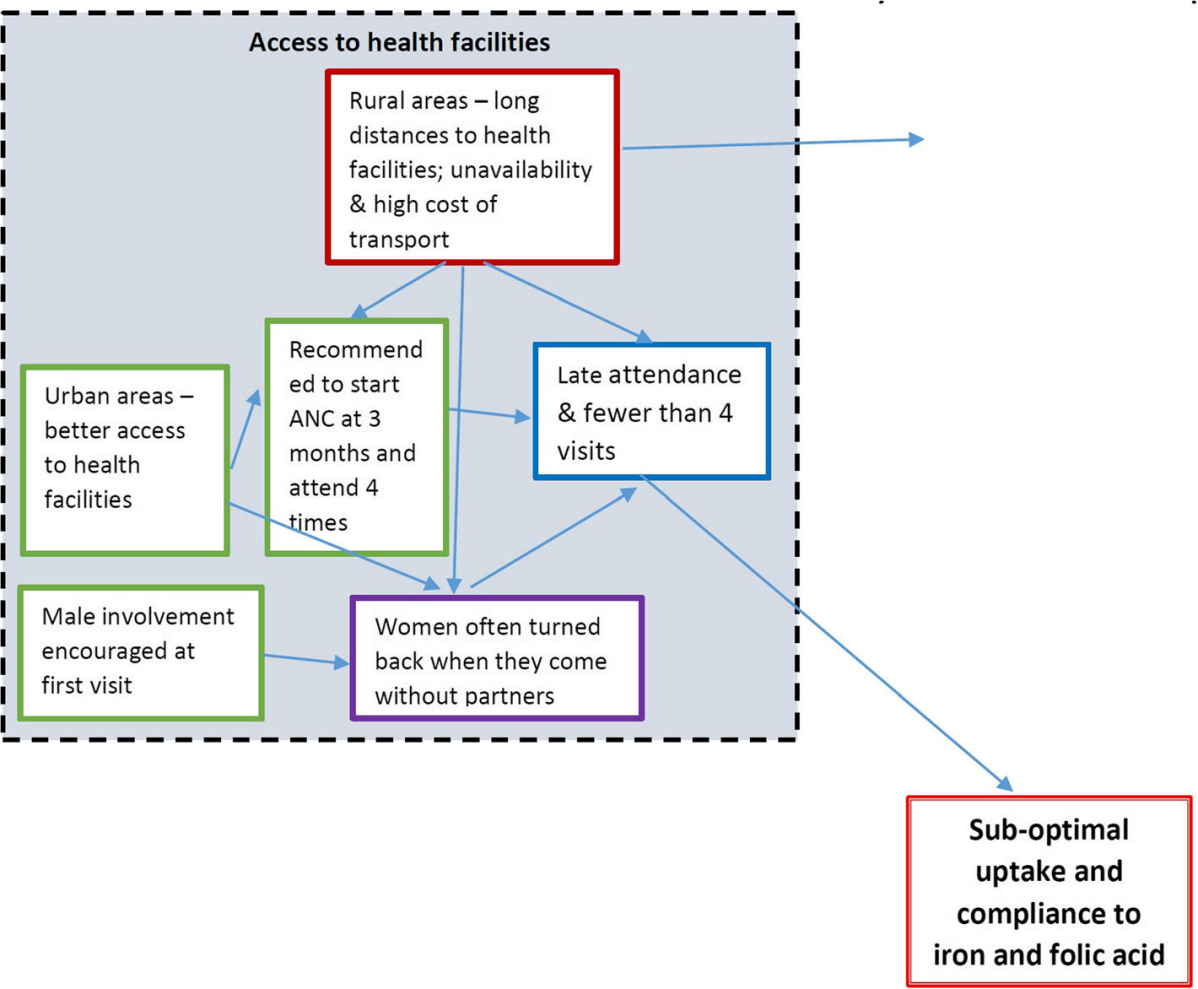
Root cause analysis of access to health facilities

*“Then the other thing that hinders us is the testing of the status so we women fear that they will find us with HIV then [we] start drinking medicine and things like that*.*” FGD participant* (Fig. 2)

### Women’s beliefs & attitudes

#### Information gaps

The main source of messages was health facilities and some had heard from community volunteers or the media, but only in very few circumstances. Community health workers presented another avenue for reaching women, especially in hard to reach areas. But these may not be present in far flung areas and even when present may not deliver appropriate messages due to lack of general training on iron and folic acid. Safe Motherhood Action Groups (SMAGs) were one group of community volunteers that were helping to disseminate messages about pregnancy.

*“Because we have tried to form the [SMAGs] groups where we teach these group members to help us teach the women in the community. We only have few SMAG groups so most of them just hear it from here [health facility].” Nurse KI*

The study noted the limited understanding on the benefits of taking and risks of not taking iron and folate supplements. These were barely understood by women beyond the effects on “blood levels” and appetite. Additional benefits of iron and folic acid supplementation such as reducing birth defects, reducing risk of eclampsia [25] and preventing stunting in children below 2 years of age [22], needed to be expanded and emphasised in order to promote the uptake and compliance to iron and folic acid. These information gaps around ANC and more specifically around iron and folic acid supplementation need to be addressed collectively.

Messages on malaria in pregnancy and family planning were among the messages least heard. Generally, in Zambia, women were provided with presumptive malaria treatment not only to prevent malaria but also to reduce the anaemia associated with malaria infection. Considering that less attention is paid to explaining the implications of malaria and family planning on anaemia, it is important to develop modules and IEC materials to be used at health facilities. These should include comprehensive information on iron and folic acid supplementation as well as family planning to adequately support women.

#### Misconceptions and fears

Misconceptions and fears around ANC generally and iron and folic acid specifically were found amongst women due to the cited information gaps. When asked why some women did not come for ANC, participants from FGDs indicated that it was “because they did not have knowledge and information on the importance of antenatal clinic”. Sometimes women just did not know what to expect when they came for ANC and thus, they were likely to get information from the wrong sources.

*“Yes, there are many pregnant mothers who don’t come to the clinic due various reasons. Some don’t come because they fear that the nurses may scold them for not having a maternity dress. Others fear that hospital/clinic staff may chase them for not coming to register on time.” Nurse KI*

Due to this limited knowledge by some women, misconceptions exist about iron and folic acid supplementation. One such misconception indicated that taking supplements resulted in big babies and increased the likelihood of giving birth by caesarean section.

*“I have heard some people say that these drugs cause the unborn baby to grow big and may end up giving birth through an operation but those with knowledge about the good effects of these drugs say it [builds] enough blood and energy levels.” FGD participant*

Still a few believed that the drugs were only for those mothers who were unwell and not needed when in good health.

*“Others say that they don’t need to take medicine for blood because they are not sick, unless if they are sick that’s when they need to take medicine for blood and vitamin.” Nurse KI*

Most mothers in the study knew that iron tablets were meant to help with increasing the amount of blood in their bodies, especially in preparation for delivery.

*“The massages talked about the importance of taking the red tablets [iron] as they help or prevent us from being anaemic while the yellow tablets [folate] encourage us to eat.” FGD participant*

Increased appetite was a recognised side effect of taking folic acid. However, for most of the women, that was understood to be the main purpose for taking it. Thus, in some cases where there was not enough food, the women did not take the supplements for fear that they would have no way to satisfy their increased appetite. This has implications for compliance to folic acid.

*“We stop taking these tablets because the hunger is too much to bear…especially in the night.” FGD participant*

*“Others are afraid of having appetite because they do not have a lot of food, so they pack the medicine.” FGD participant*

Amongst health staff who teach women at ANC the emphasis on folic acid was on prevention of birth defects, but anaemia was sometimes not discussed, as different untrained cadres did the teaching due to staff shortages. Such cadres included community volunteers who might not have been equipped with the required knowledge. However, health staff reported endeavouring to address iron and folic acid in all their teaching sessions to decrease misconceptions and fears. The mothers interviewed also reported that iron and folic acid were covered as topics in the teaching, mainly when discussing nutrition.

**Fig. 3:**
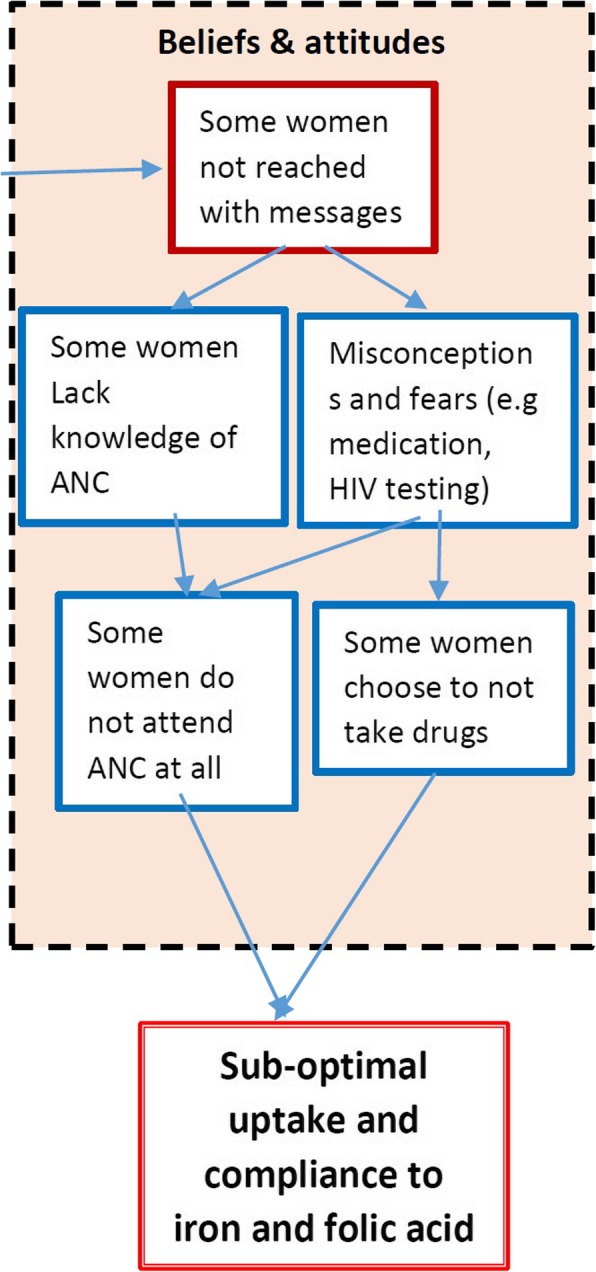
Root cause analysis of women’s attitudes and beliefs

*“…we make sure that we don’t do a session without talking about the iron and [folic acid] because they have a misconception or misinformation which we are trying to eliminate by all means by educating them they say if they take those supplements then the baby will grow the head, the head will be so big such that it will not be possible for them to deliver and spontaneous delivery so we tell them that it is not true.” Nurse KI* (Fig. 3)

### Practices

#### Forgetfulness to take medication

For those that took the iron and folic acid supplements, who were the majority, forgetfulness to take the medication was an important factor in relation to compliance. Regarding taking all the tablets given, the majority (80.5%) of the women reported having taken all the tablets offered to them. Focus group discussions however revealed that compliance was still a challenge. Daily intake was a very demanding task. Thus, it was not surprising that many women forgot to take the supplements. Inadequate support from partners contributed to this as there was no one to remind the woman to take the medication. Male partners who attended ANC with their spouses were said to be of help in this regard at times, but not always.

#### Inadequate monitoring mechanisms

There was overall, no reliable mechanism in place to monitor compliance to iron and folic acid once the woman left the health facility. Drugs such as anti-malarial and de-worming medication were taken right at the health facility before the woman goes home and thus this was easier to monitor. However, drugs taken at home present a challenge for monitoring purposes. Drug counts at the health facility and community health workers presented two possible mechanisms that might be utilised in the communities to support women to take the supplementation. Health workers were aware of the need to strengthen this monitoring mechanism. For example, some health facilities started using SMAG groups to check on mothers within the community and see if they were taking the supplements. One nurse narrated the involvement of the SMAGs in supporting women at the community level.

*“So we also involved the SMAGS to inspect and see that they are taking folic and the iron supplements.” Nurse KI*

However, at the time of the study, the outcome of this initiative was unclear. The role of male partners and other relatives was also appreciated and encouraged in order to allow women to be reminded to take the supplements. Children were found to be useful agents in this regard, sometimes reminding their mothers to take the supplements. The health staff explained:

*“We also need to involve them [children] as we educate them, we also need to involve the relatives and also the spouses so that they are monitored that they are really taking the supplements.” Nurse KI*

**Fig. 4:**
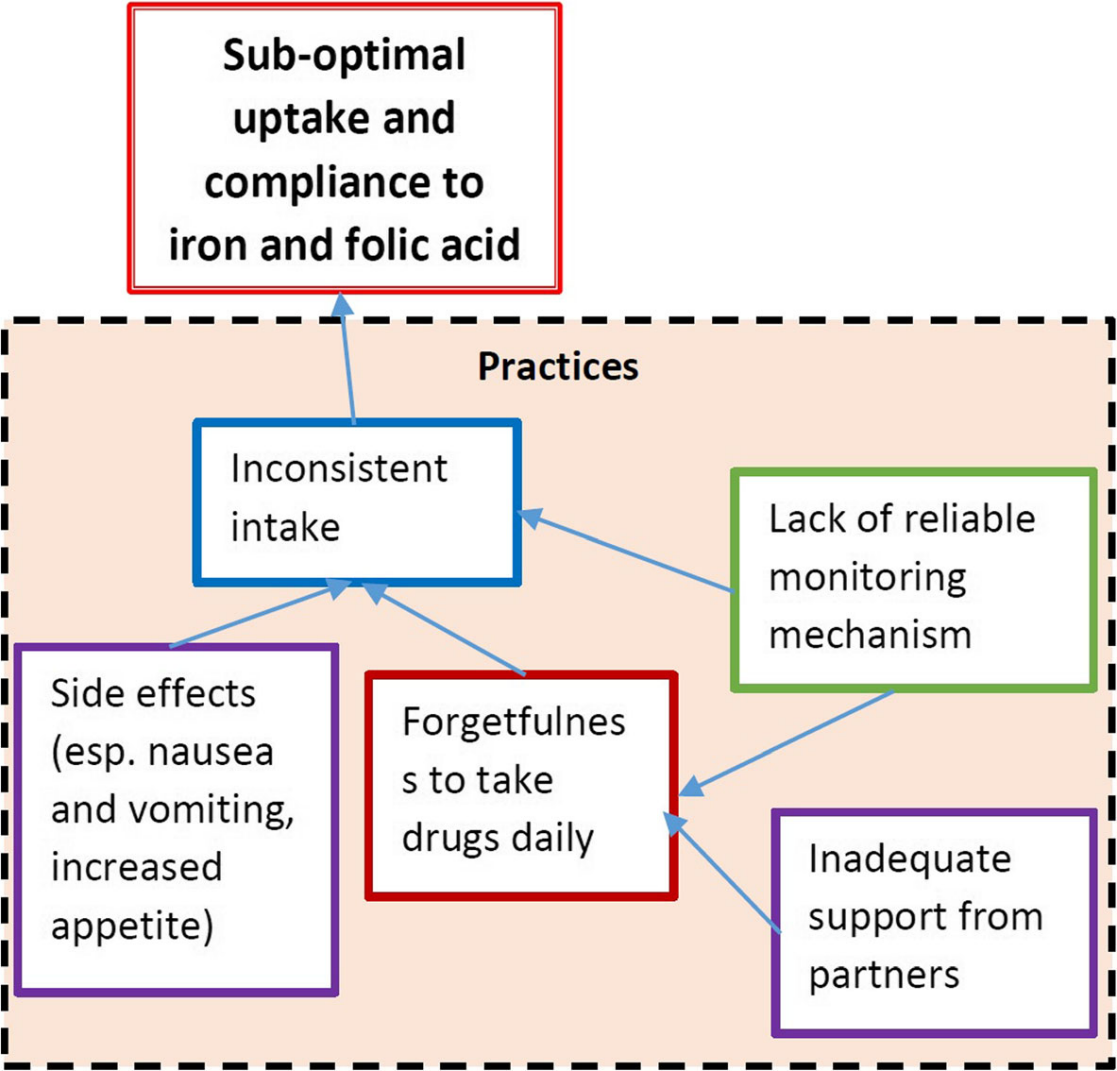
Root cause analysis of practices

Other challenges associated with non-compliance to taking iron and folic acid included side effects such as nausea and vomiting. Some women did not continue taking the drugs or took them inconsistently due to these side effects (Fig. 4).

### Health worker numbers, knowledge, attitudes and practices

#### Inadequate and inconsistent messages during ANC around folate and iron

There was no standardized training around delivery of antenatal messages across different health cadres. Most health facilities were understaffed and therefore depended on different cadres to provide teaching during antenatal clinics. Nurses were responsible for conducting such sessions, but when busy this was left to inadequately trained cadres such as community health workers or SMAG members. There was generally inadequate time dedicated to delivering messages about antenatal care, iron and folic acid. Involving men in the first visit put further increased pressure on staff. Teaching sessions were conducted quickly as the men were presumed to be busy and preferred to leave the antenatal session hastily. Some health facilities reported having a fast-track route for women who came with their partners so that they did not take too long at the health facility.

*“Usually these messages if we are to talk about them in detail it could take a lot of time. With the male involvement, when we keep the men for a long time they complain. So, what we talk about are things not really in details partly to avoid keeping them for a long time. We don’t really go much into detail.” Nurse KI*

Furthermore, health workers utilised mainly the first visit to educate mothers and their partners on all matters related to their pregnancy. Subsequent later visits were rarely utilised to educate the women and their partners. This presented a lost opportunity, particularly given that women were required to attend ANC only four times during their pregnancy. Further hindering the teaching process was a lack of IEC materials to use during such sessions. Only one health facility visited had IEC material in the form of a chart that could be used to teach the different relevant aspects of pregnancy. This IEC material was given to them by a donor funded project. There were no government produced IEC materials on antenatal teaching available at the health facilities visited. This made it difficult to deliver consistent and correct messages across health facilities and health cadres.

Consequently, the messages also varied in detail with some women receiving more detailed information than others. This resulted in having differing knowledge levels. Literacy levels of women were another factor to be taken into consideration when delivering antenatal messages and messages on iron and folic acid. Urban mothers overall seemed more knowledgeable about iron and folic acid than rural mothers during FGDs.

**Fig. 5:**
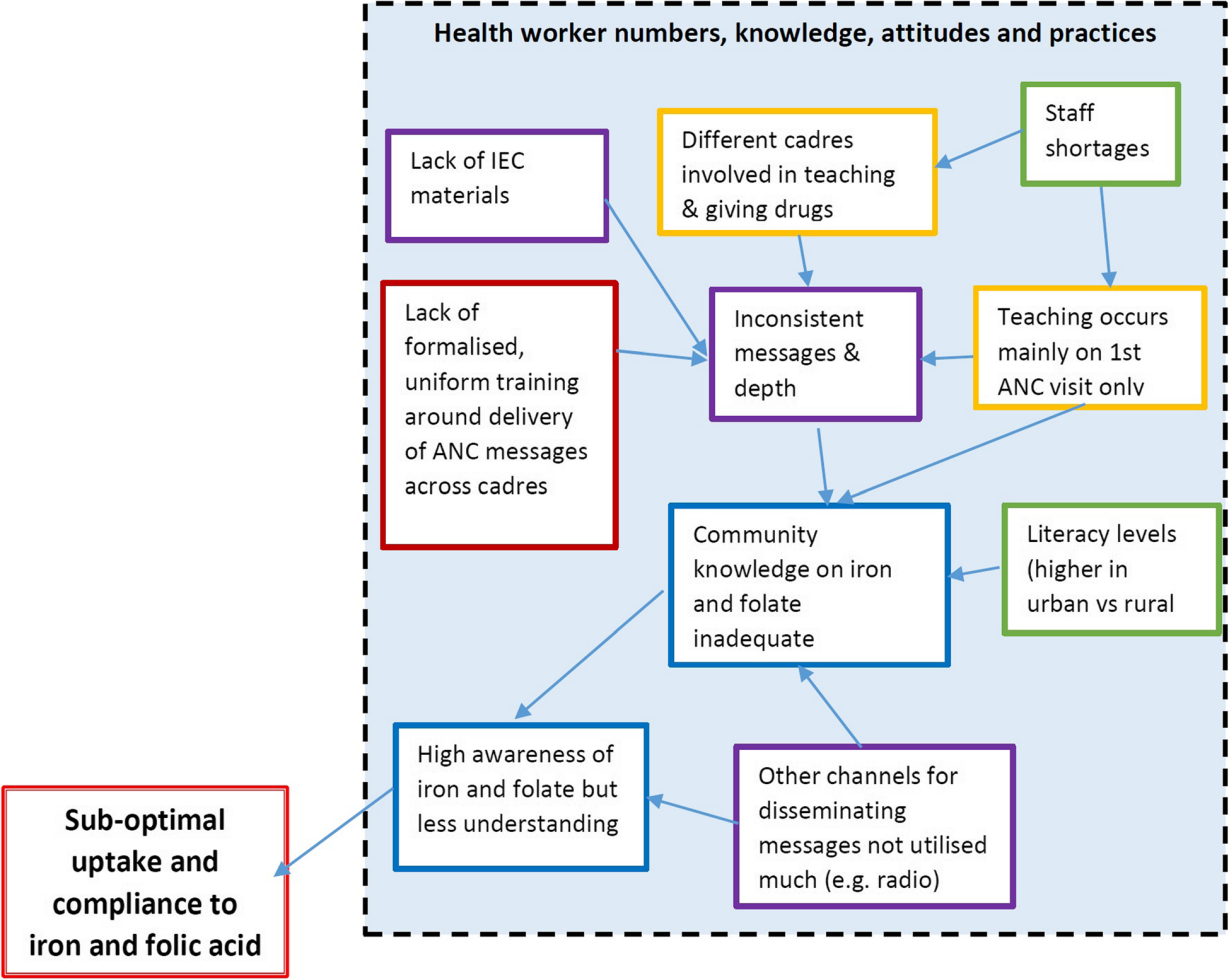
Root cause analysis of health worker practices

The majority of both men and women surveyed reported that they had understood the messages very well. However, only about 55% of women and 43% of men surveyed, reported being given a chance to ask questions all the time. This shows the inadequacy of the time spent during these teaching sessions and that more emphasis should be given to providing thorough information and allowing understanding of the messages (Fig. 5).

### Availability of iron and folic acid

#### Adequate supplement stocks and free provision

Stocks of drugs were adequate at most health facilities sampled. Stock-outs were not a major factor affecting uptake and compliance to iron and folic acid. Nearly all women surveyed obtained their iron and folic acid tablets, mainly the coated type, from the health facility at no fee. Therefore, the price of the drugs was not a factor with regard to uptake and compliance. A few health facilities reported stock-outs prior to the study. They also indicated that this was uncommon as health facilities usually had a good supply from the government.

#### Mismatch between frequency of visits and drug allocation schedules

Women in the study generally did not attend ANC on a monthly basis since only four visits were recommended during the entire pregnancy. Nonetheless, many did not achieve even these four visits. In most cases, when a woman visited ANC, she was given a month’s supply of both iron and folic acid. This meant that she would run out of these supplements before the next antenatal visit if she took them daily as instructed. This practice had implications for compliance to iron and folic acid.

*“…they advise us to come and collect [when we run out of medication] so it’s all up to you… but most of us when the medicine finishes we just wait for the next visit.” FGD participant*

There were attempts to utilise community volunteers, particularly SMAG groups, to distribute iron and folic acid to pregnant women in communities. However, this was often hindered by having few numbers of volunteers as well as transport challenges to reach household in the community. Some SMAG members were equipped with bicycles and others were not.

#### Lack of awareness of alternatives

Alternative sources of the drugs such as local chemists and pharmacies were very rarely utilised by the mothers as were alternative preparations like syrup. Most mothers were unaware that they could obtain iron and folic acid in syrup form as the health facilities did not stock these routinely and did not teach about them in antenatal sessions. In health facilities, it was taken for granted that access to iron and folic acid was only feasible at health facilities. There was no effort made to ensure that pregnant women were aware of other sources for the supplements.

**Fig. 6:**
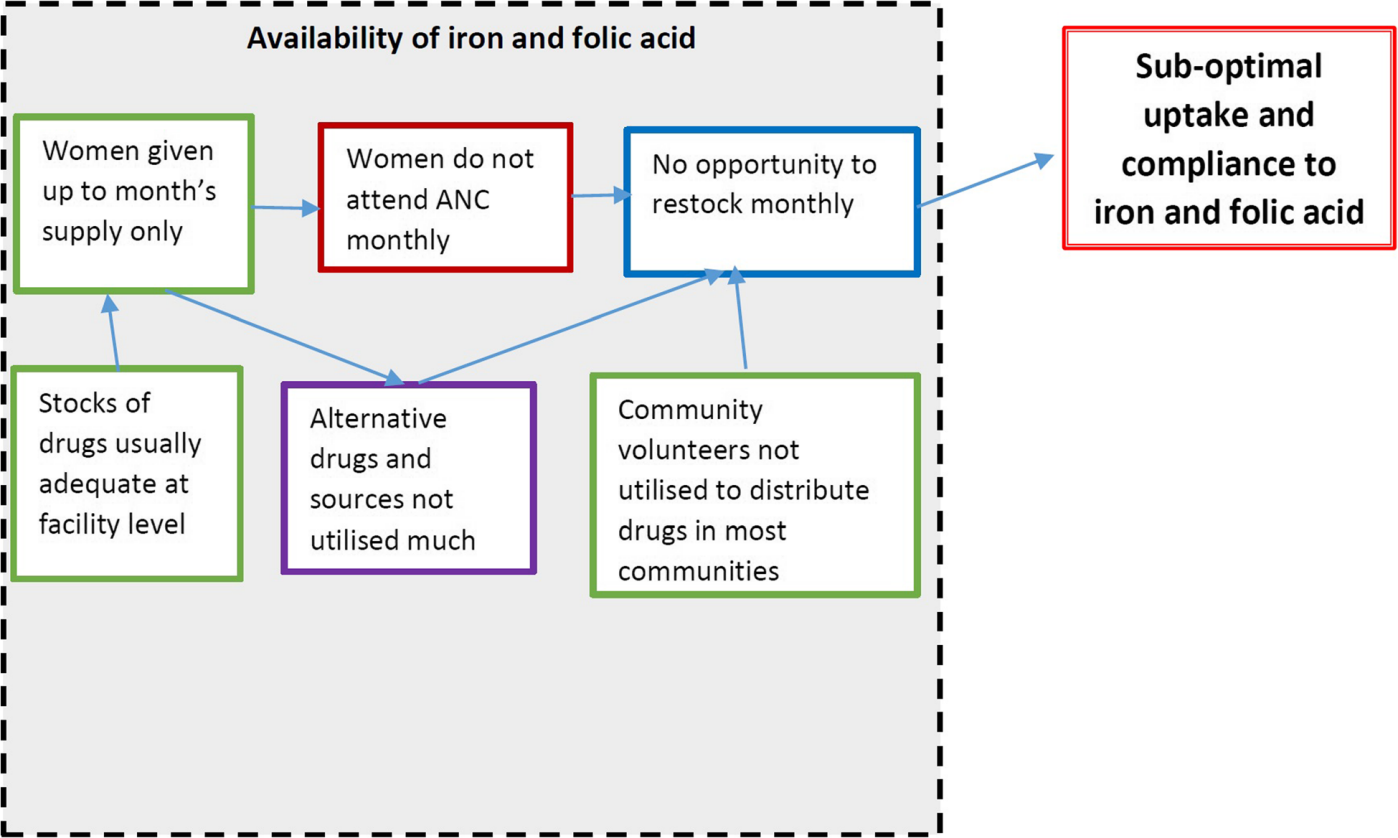
Root cause analysis of availability of iron and folic acid

The study findings should be interpreted with reference to several limitations. These include that it was not a nationwide sample, although the provinces, districts and health facilities selected overall would be considered representative of the country. Secondly, as the findings were self-reported and considerable time may have elapsed between pregnancy and time of this survey, recall bias as well as political correctness and courtesy bias are a possibility. A third limitation was that levels of anaemia were not assessed given that no biological samples were collected and not all women interviewed were pregnant at the time (Fig. 6).

## Conclusion

This study highlighted challenges that women encounter in order to receive iron and folic acid supplementation. Both uptake and compliance were found to have been sub-optimal. While male involvement may be a supportive factor, access to health care facilities, women’s and health care workers’ knowledge, attitude and practices were important factors that influenced early uptake and compliance. It is important to improve messages delivered to women to encourage early initiation of antenatal care and required number of visits, as well as improve uptake and compliance to iron and folic acid supplement. There is also need to standardise teaching materials and utilise available channels to disseminate messages around antenatal, iron and folic acid supplementation.

## Data Availability

The data files have been deposited on Springer Nature Research Database and are publicly available.

## Abbreviations

ANC: Antenatal Care
CSO: Central Statistical Office
FCE: Full Country Evaluation
FGD: Focus Group Discussion
IEC: Information, Education and Communication
KI: Key Informant
KII: Key Informant Interview
MOH: Ministry of Health
NFNC: National Food and Nutrition Commission
RCA: Root Cause Analysis
SEA: Standard Enumeration Area
SMAGS: Safe Motherhood Action Groups
SUN: Scaling Up Nutrition
WB: World Bank
WHO: World Health Organisation
